# Sources of Variation in a Two-Step Monitoring Protocol for Species Clustered in Conspicuous Points: *Dolichotis patagonum* as a Case Study

**DOI:** 10.1371/journal.pone.0128133

**Published:** 2015-05-26

**Authors:** Virginia Alonso Roldán, Luisina Bossio, David E. Galván

**Affiliations:** 1 Centro Nacional Patagónico, Concejo Nacional de Investigaciones Científicas y Técnicas, Puerto Madryn, Chubut, Argentina; 2 Universidad Nacional de la Patagonia San Juan Bosco, Puerto Madryn, Chubut, Argentina; Peking UIniversity, CHINA

## Abstract

In species showing distributions attached to particular features of the landscape or conspicuous signs, counts are commonly made by making focal observations where animals concentrate. However, to obtain density estimates for a given area, independent searching for signs and occupancy rates of suitable sites is needed. In both cases, it is important to estimate detection probability and other possible sources of variation to avoid confounding effects on measurements of abundance variation. Our objective was to assess possible bias and sources of variation in a two-step protocol in which random designs were applied to search for signs while continuously recording video cameras were used to perform abundance counts where animals are concentrated, using mara (*Dolichotis patagonum*) as a case study. The protocol was successfully applied to maras within the Península Valdés protected area, given that the protocol was logistically suitable, allowed warrens to be found, the associated adults to be counted, and the detection probability to be estimated. Variability was documented in both components of the two-step protocol. These sources of variation should be taken into account when applying this protocol. Warren detectability was approximately 80% with little variation. Factors related to false positive detection were more important than imperfect detection. The detectability for individuals was approximately 90% using the entire day of observations. The shortest sampling period with a similar detection capacity than a day was approximately 10 hours, and during this period, the visiting dynamic did not show trends. For individual mara, the detection capacity of the camera was not significantly different from the observer during fieldwork. The presence of the camera did not affect the visiting behavior of adults to the warren. Application of this protocol will allow monitoring of the near-threatened mara providing a minimum local population size and a baseline for measuring long-term trends.

## Introduction

Estimations of abundance are fundamental in ecology and conservation to answer a wide range of questions. Often scientists and decision makers need to compare abundance through space or time, and for this purpose, it is important to estimate possible sources of variation in the probability of detecting an individual [1]. Several methods to estimate abundance that cope with imperfect detection have been developed, but these methods make several assumptions. Mark-recapture methods require live-trapping and marking of animals or naturally recognizable individuals for camera-trap applications of the models [2]. Distance sampling and Random Encounter Models require a minimum number of encounters and random design [3, 4]. These conditions are difficult or impossible to meet in rare or elusive species that are also difficult to catch or when individuals are not consistently recognizable.

Random designs yield extremely low encounter rates are observed in species that are found associated with a particular feature on the landscape, such as rocky outcrops for reef fishes (*Pseudopercis semifasciata* [5]), cliffs for mountain vizcachas (*Lagidium viscacia* [6]) and the Andean condor (*Vultur gryphus* [7]), or conspicuous warrens for maras (*Dolichotis patagonum* [8, 9]) and plains vizcachas (*Lagostomus maximus* [10]). In these cases, counts are commonly made by making focal observations close to the feature where animals concentrate (e.g. *Suricata suricatta [11]*, *Vultur gryphus* [12], reef fishes [5, 13], *D*. *patagonum* [14, 15]). Focal observations provide an index that is restricted to specific points in space so additional information is needed to obtain density estimates for a given area. As an alternative, density can be estimated by searching for sign and estimating occupancy rates of suitable sites combined with independent estimates of the average number of individuals aggregated around each sign [16]. We propose a two-step protocol in which random sampling designs are used to search for signs and continuously recording video cameras are used to perform abundance counts at the points where animals are concentrated. However, to avoid confounding effects, we incorporated several factors that influence detectability and introduce potential bias in abundance estimation and inference.

In this context, the detection probability and other sources of variation that must be estimated have at least two components: one related to the searching for sign and another related to counts of animals using video cameras at the focal observations. Sources of potential bias in abundance estimations using video cameras could be related to restricted viewpoints [17], changes in behavior of target individuals caused by the presence of the device [18] and temporal variation in detectability due to animal movement [13]. However, these biases could be minimized due to resource influence in the case of animal counting around shelters or other resources that concentrate individuals. The camera may record most of the individuals if: it covers the main resource influence area; behavior is normalized when individuals tend to accept a new static object near shelters or food sources [19]; and the sampling schedule is designed to include patterns of resource use, which would reduce temporal variation [13, 20]. In addition, although using continuously recording video cameras avoids the false negative bias related with triggers, the influence of the length of a sampling period over detection or counting persists. Unfortunately, perfect detectability should not be assumed even if all these sources of bias could be controlled or minimized. Detectability may be estimated using the proposed approach by repeated observations [1].

The mara, an endemic mammal of the Argentine semi-desert, was classified as ‘Near Threatened´ according to the 2008 IUCN Red List assessment due to population decline [21]. The reported trend was based on expert knowledge according to field observations given the absence of systematic data aimed to describe population abundance and direct abundance estimates. This species is a good model to apply the proposed two-step protocol and to assess sources of variation because of its communal breeding behavior and the distribution pattern associated with this behavior. The same behavior also makes it impossible or impracticable to use well-established methods. Maras form long-term monogamous couples that spend most of the year dispersed over wide areas with a radius of up to 2000 m around warrens, avoiding other couples [8, 9], and this dispersion makes them difficult to detect. Thus, encounter rates are too low (Section A in S1 File) to make accurate estimations using distance sampling, and the assumption of independent detection events could be violated because of poorly defined clusters around warrens [4]. Mark recapture models [2] are not applicable because live-trapping of maras results in a high mortality rates [22] and it is not possible to recognizing individuals visually. On the other hand, couples stay close to communal warrens during the breeding season, from August to December where they can be more easily counted.

Warrens are tunnels in the ground where pups of several couples spend the night and most of the day, using it as a shelter to avoid predators. In contrast, adults do not actively use the warrens for shelter but spend part of the day next to the warren nursing their pups or on alert while pups play and feed on vegetation [14]. Warrens are easier to detect than maras and could persist, even when not actually in use by maras, causing false-positive detection [16]. Moreover, the number of maras associated with a warren is variable among warrens and through time. Warrens accommodate pups from one to more than ten couples [8, 9, 15]. In addition, a breeding pair visits the warren at least once a day to attend to their pups [14], but because it is not possible to recognize individuals, repeated visits cannot be differentiated from new couples approaching. Thus, the number of adults near warrens could vary through time during the day depending on how many couples are visiting their pups together and also because some environmental circumstances are perceived as threats that may make adults leave the warren.

Previous studies on the ecology and behavior of maras have utilized direct counts made by an observer to quantify the number of adults associated with studied warrens [9, 14, 15]. In recent studies, the observers have been replaced by surveillance video cameras that register the activity around warrens and allow researchers to count the maximum number of adults visiting the warren together and the number of resident pups of different age classes [8]. The use of video cameras provides significant logistical and methodological benefits because it makes it possible to observe several warrens at the same time with no need for multiple observers [23], reducing fieldwork costs, avoiding bias due to differences among observers and diminishing disturbances in the study area [24–26]. As mentioned above, this technique might be biased due to i) the detection capacity of the camera, ii) the perception of the camera as a threat, and iii) variation in the timing and frequency of adult visits.

The aim of this study was to evaluate possible bias and influence of several factors over detectability when applying the two-step protocol using the mara as a case study. Specifically, our objectives were to quantify detectability of warrens performing line-transect samplings within defined plots. We also estimated the bias related to false positive detection due to misidentification or the persistence in the field of abandoned warrens that have no associated couples (non-active warrens). We evaluated the detection capacity of our camera against an observer in the field. This way we were able to evaluate changes in visiting behavior due to the presence of the camera. Finally we evaluated and quantified temporal variation in the number of adults. Temporal variation in the detection probability could be related to: i) the length of sampling period, ii) the diurnal trend in visiting dynamics and iii) the inclusion of inactive warrens in estimations.

## Materials and Methods

### Study site

**Fig 1:**
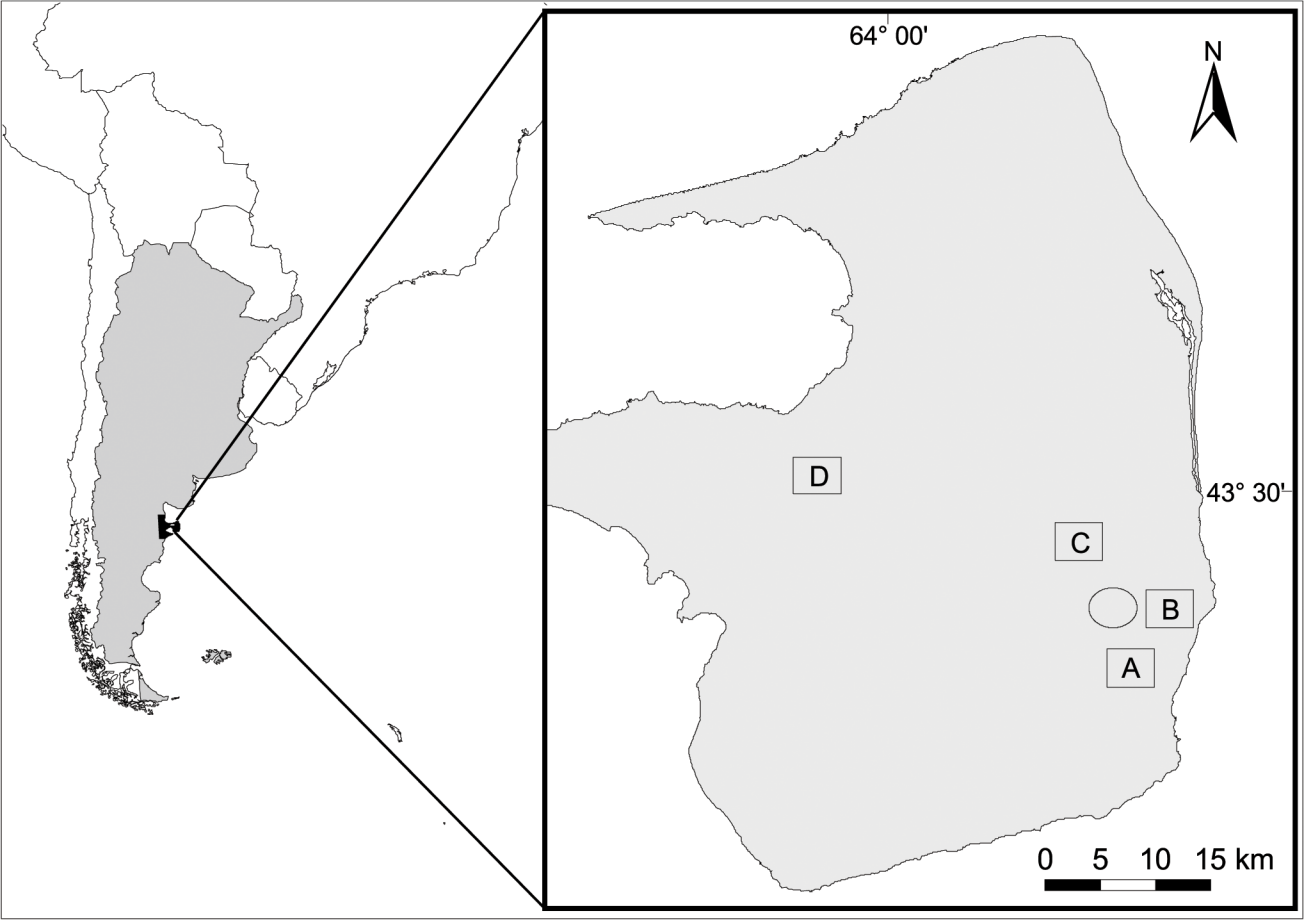
Study site location. Map showing the location of Península Valdés (Argentinean Patagonia) in South America (left panel) and sampling sites (right panel). Rectangles show the location of the four 2000-ha areas used to search for warrens and estimate detectability and false positive bias, while the circle shows the location of the 12 warrens surveyed to quantify bias in the counts. A and B indicate the sites placed in grassland, C and D the sites placed in shrub-grass. We have created the image ourselves using ArcView 3.2.

The study was carried out in Península Valdés (Argentinean Patagonia), a 4,000 km^2^ provincial protected area declared a UN World Natural Heritage Site (Fig 1). Península Valdés currently has the IUCN category of ‘managed ecosystem’ and consists of private properties where sheep ranching is the main productive activity. Within Península Valdés, vegetation structure varies but can be described by three main landscape configurations: shrubland, shrub-grass mosaic and grassland.

### Warren detection

We used volunteer observers to assess the detectability of warrens by applying a line transect sampling design within a given area (searching plots). We searched for warrens while walking along 20 parallel transects that were 5 km long and 200 m apart in four 2000-ha areas in Península Valdés (42.48°S 62.12°W, 42.55°S 63.76°W, 42.61°S 63.64°W and 42.67°S 63.69°W). Volunteer observers were briefly trained in the field before walks by showing them warrens, feces and footprints of mara and by comparing warrens with peludo (*Chaetophactus villosus*) burrows because these structures can be easily confused. We selected the size of our sampling area in proportion with the home range size (193 ha [9]) so it was large enough to possibly contain 10 warrens or more. Study sites represented the two main contrasting landscapes within the protected area with suitable habitat characteristics for mara [8]: shrub-grass mosaic and grassland (Fig 1). We placed study sites where we knew that the species was present, based on knowledge from local people and previous visits, because i) we were interested in sources of variation in the counting of warrens rather than species occurrence, and ii) random points within Península Valdés produced few warren encounters with high cost (Section B in S1 File). After the line transect survey, but in the same period between reproductive seasons to avoid the digging of new warrens (closed population), the author (VAR) surveyed the areas intensively, searching for undetected warrens, walking in several directions through located warrens, walking in several directions within areas where warrens were not found and visiting with local people in sectors where they had usually seen maras while working. In addition, we visited each point marked by volunteer observers to check if it was a mara warren or a case of misidentification. We calculated the detectability of warrens based on the mark-recapture concept and double sampling approach [1] applying the following equation:

$$\beta ={\text{m}}_{2}/{\text{n}}_{2}$$ (1)

where β is detectability, m_2_ is the number of warrens “marked” during the line transect survey and n_2_ is the total number of warrens known after the intensive second survey (i.e. double sample). We also calculated the error associated with each observer because of misidentification as the proportion of wrongly marked warrens over the total number of marked points.

We surveyed identified warrens through the reproductive season to obtain the proportion of active warrens in order to evaluate bias related to detection of false positives of abandoned warrens. We set continuously recording video cameras for one day every 15 days in 27 warrens and repeatedly visited the remaining warrens searching for changes in signs of activity, such as recently removed soil at the warren entrance, new footprints or feces [15]. Camera features and settings were the same used to assess counting (see next section for details).

### Counting adults

#### Surveillance setting

Twelve mara warrens were surveyed during the middle of the mara breeding season (between 22 October and 10 November in 2011) to assess possible bias and the effects of several factors on the detectability of adult mara using continuously recording video cameras. These warrens were outside the searched areas described above, were previously known, were active in previous reproductive seasons and were easily accessed. All warrens were located within a private sheep ranch in the southwest of Península Valdés (Patagonia, Argentina; Fig 1, 42.62°S 63.71°W), where the predominant vegetation is a shrub-grass mosaic of *Chuquiraga avellanedae* and *Stipa tenuis* [27].

**Fig 2:**
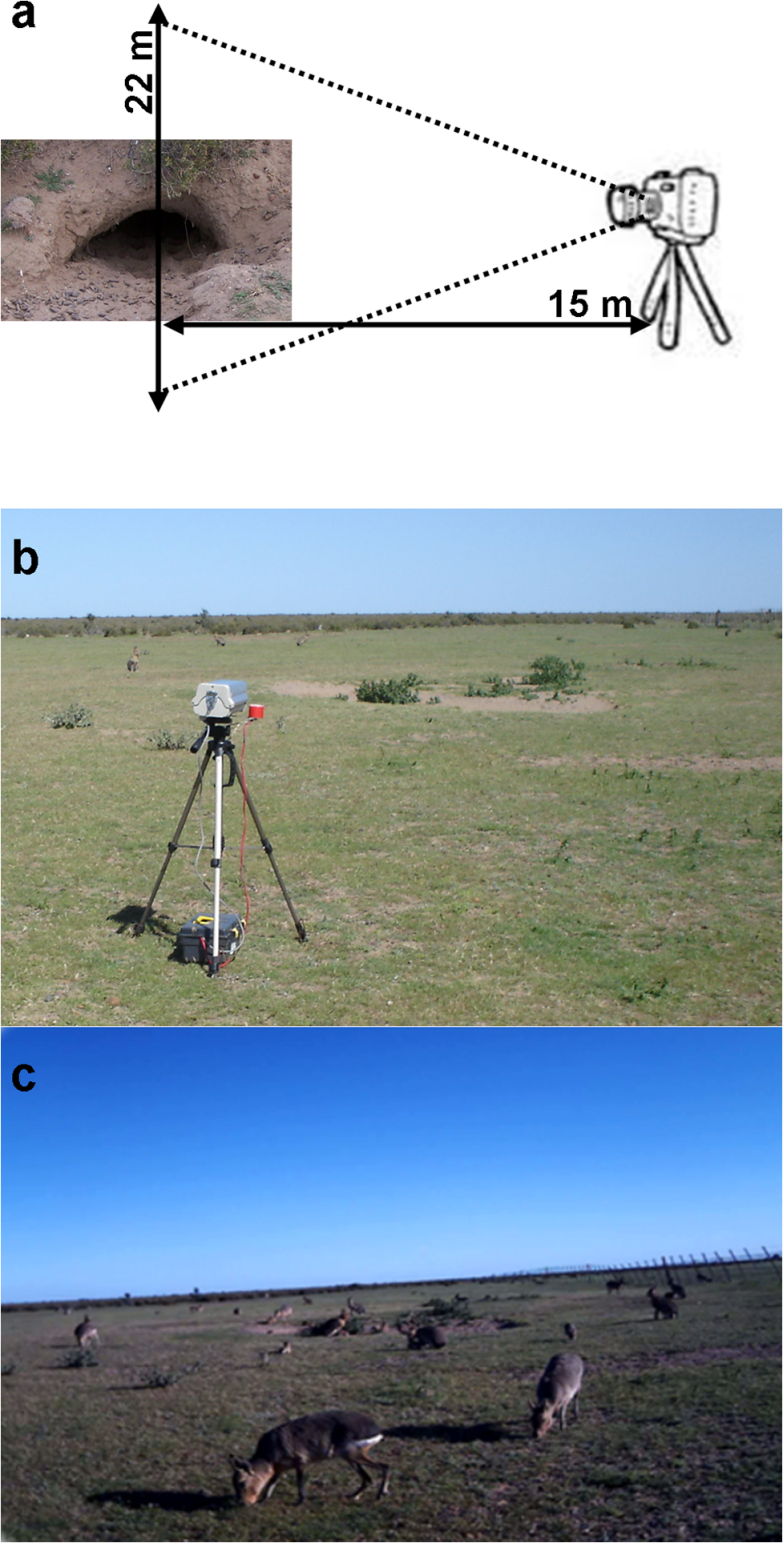
Camera setting. Panel a shows the distance between the camera and warren and the surveyed area given camera’s lens aperture. Panel b shows an example of a camera setting in a shrub-grass landscape. Panel c shows a snapshot of a video recorded in a shrub-grass landscape.

Each warren was monitored using a surveillance camera with 2 MPixels (Vivotec, IP7160) placed approximately 15 m from the entrance (Fig 2) to register the activity for 13 hours per day (7–20 h). The cameras were powered by 12 V batteries with a photocell that cut off power at night, saving energy and memory storage capacity for daylight hours when maras are active [28]. Surveys were stored in one minute long mp4 format videos.

#### Detection capacity of camera vs. direct counting

We compared the maximum number of adults recorded simultaneously by direct observation and by the camera within the same period of time (7:30–11:30) to evaluate the accuracy of counting from video with respect to direct counting. Direct observations were conducted from a platform located more than 50 m from the warren that was simultaneously monitored by the camera. The comparison was performed by means of a paired Wilcoxon’s signed-rank test.

#### The effect of a camera on mara behavior

We recorded mara behavior when cameras were present and when they were absent. First, to evaluate if the camera was inhibiting the approach of maras to the warren, we used direct observation to count the number of maras distant from and close to the warren using a circle centered at the warren with radius equal to the distance between the warren and the camera (approximately 15 m) as a reference. Second, to evaluate if the camera was perceived as a threat by maras, we counted the number of alert maras (head up and looking around) and relaxed maras (resting, feeding or interacting with pups). We built contingency tables to compare the number of distant/close and alert/relaxed individuals with and without the camera present by means of Χ^2^ tests, discarding data from warrens with null observations and applying the Yates’ continuity correction when the number of observations was less than 200 [29].

#### Temporal variation

The temporal variation in the number of adults detected by the camera was evaluated by surveying each warren for three days (not always consecutive). Given that the videos were stored in one-minute files, the raw data consisted of the maximum number of adults recorded simultaneously in each minute. We used the videos to differentiate active warrens, such as the ones in which resident pups were registered, from inactive warrens that have no couples or pups associated with them but could be visited occasionally by non-resident individuals.

To evaluate the effect of the length of the sampling period on detection probability, we examined the data set taking the maximum number of adults observed in random time-frames of increased duration by 10 minutes, from 10 to 770 minutes. We took different starting points at random within the data set from each sample unit (a given warren in a given day) for the 77 possible time-frames, performing one thousand iterations of that process. We expressed the maximum obtained in each time-frame and iteration as a proportion of the maximum registered in the corresponding warren during the three days of surveillance. The mean of this proportion was plotted against the length of the sample period to identify the time-frame where the calculated proportions reached within 0.05 of the asymptotic value (i.e., shorter sampling period with highest accuracy). All the calculations were performed using R statistical software [30].

To evaluate if there is a diurnal trend in the visiting dynamics, we compared the maximum number of adults per minute detected by the camera during the morning (7–11 h), the middle of the day (11–15 h) and the afternoon (15–19 h). We fitted a linear mixed model, including time of day as a predictor and the warren and the date in which the observation was made as hierarchical random factors to account for the variation due to heterogeneity among experimental units. We used a temporally structured variance term to address autocorrelation. We considered only the active warrens because registration of non-resident individuals could mask visiting patterns of resident individuals.

Finally, we quantify the variation in estimates of the number of adults associated with a warren due to the estimations taking place on different days. We calculate the maximum number of adults registered in each day of observation in each warren and fit a linear mixed model, including the warren identity as a random factor. To evaluate the possibility that the variation was overestimated due to the registration of non-resident individuals, the model was fitted twice: once considering all warrens and again considering only the seven warrens where resident pups were registered (active warrens).

We used R statistical software [30] and the R-package nlme [31] to fit the models. In both cases, the natural logarithm of the maximum number of adults plus one was the dependent variable. Normal distribution of the error was chosen because a plot of the mean versus the variance of the response variable [32] showed that the Poisson distribution was an inappropriate choice. Given that the mixed models decompose the sources of variation due to the random effect and residual variation, we obtained estimates of the variation due to differences in warren characteristics (random factor), to temporal factors and to the detection capability of the method (residual variation). To quantify variation in biological units applicable to other estimations, we expressed it as coefficients of variation (CV).

## Results

During the line transect search with volunteer observers we found 12 and 16 warrens within grassland sites, areas A and B, respectively, and 12 and 16 warrens within shrub-grass sites, areas C and D, respectively, giving an average encounter rate of 0.14 km^-1^. The posterior intensive survey showed 6 undetected warrens in area B and 3 in each other area. Therefore, detectability was 0.8 in area A, 0.73 in B, 0.8 in C and 0.84 in D. The error associated with each observer because of misidentification was 0.47 on average, varying between less well trained observers (0.62) and highly trained observers (0.22). The error due to the persistence of abandoned warrens was 0.92 on average, given that only one of the located warrens was active in area A and B, none in C and four in D.

The maximum number of adults registered by direct observation and by the camera were not significantly different (Wilcoxon’s signed-rank test: *T* = 9, *P* = 0.21). We did not find evidence indicating that the presence of the camera affected the behavior of mara approaching the warren or that the camera was perceived as a threat by maras. The proportion of distant individuals did not show significant differences with and without the camera present in three warrens (Table 1), and it was lower with the camera present in two warrens (0.13 and 0.46 with camera, 0.73 and 0.77 without camera, respectively). Only in one warren were more distant individuals registered with the camera present (0.43 with camera and 0.13 without camera). Regarding the proportion of alert individuals, there were no significant differences with and without camera present in four warrens (Table 1), and the proportion was lower with camera present in one warren (0.37 with camera and 0.54 without camera). Only in one warren were more alert individuals registered with the camera present (0.63 with camera and 0.36 without camera).

**Table 1:** Camera effect over mara behavior.

	Proximity		Alertness	
Warren	*X* ^*2*^	*P*	*X* ^*2*^	*P*
1	0.049	0.824	3.444	0.064
2 c	5.223	0.022	0.003	0.955
3 c	0.009	0.925	2.012	0.156
4	0.424	0.515	12.672	3.00E-04
7 c	36.796	1.31E-09	0.036	0.849
12 c	8.564	0.003	7.984	0.005

Results of Χ^2^ tests based on contingency tables comparing the number of distant/close and alert/relaxed individuals with and without the camera present. The informed X^2^ statistic (*X* ^*2*^) and the associated probability (*P*) are given, and subscript c in the warren’s number indicates cases where Yates’s correction was applied.

**Fig 3:**
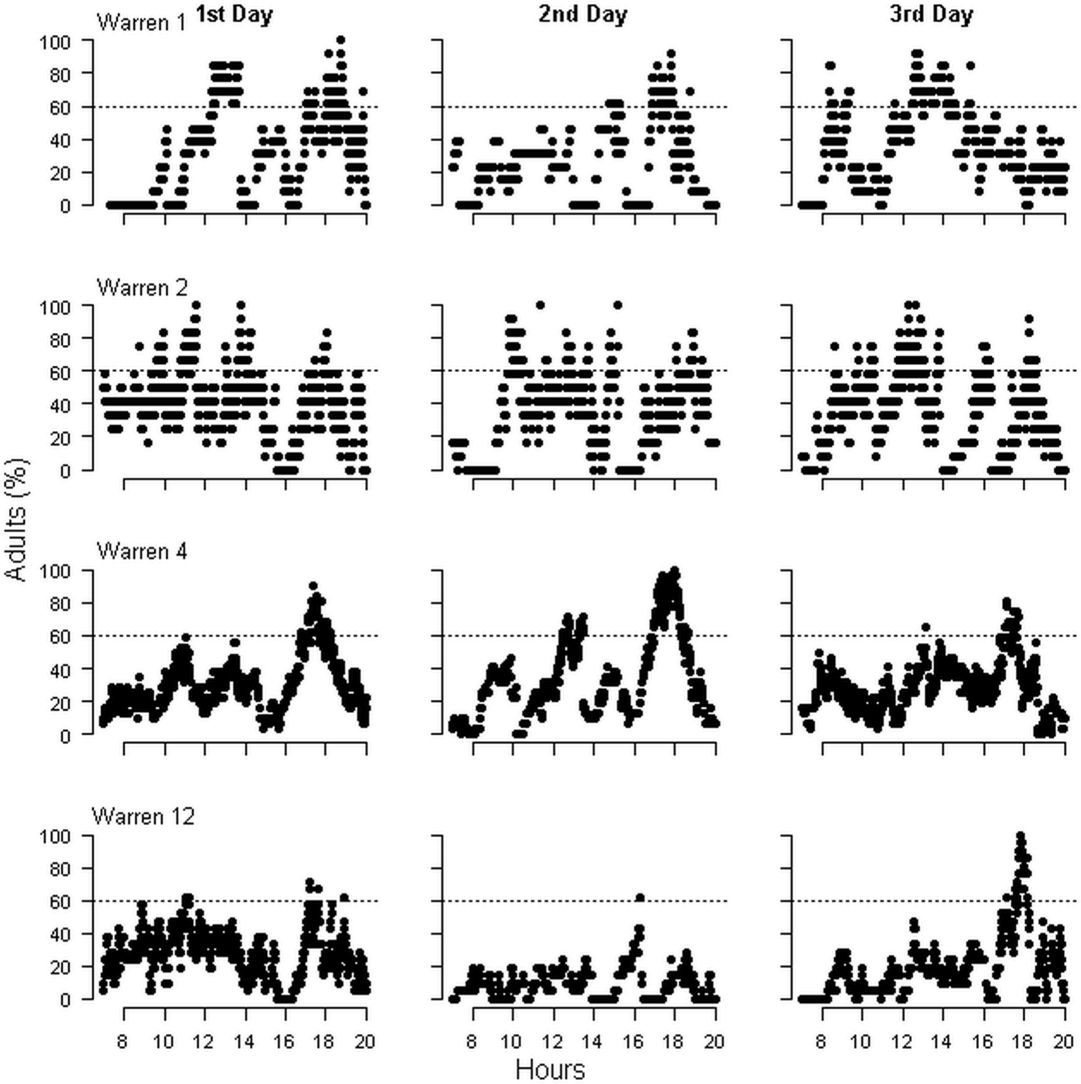
Daily variation in the number of adults registered. Charts show examples of the visiting dynamic of adults to the warren during the three surveyed days in four warrens where resident pups were registered. The maximum number of adults registered within 1-minute intervals is expressed as percentages of the absolute maximum for each warren during the entire study. The dotted line indicates 60% of the maximum number.

**Fig 4:**
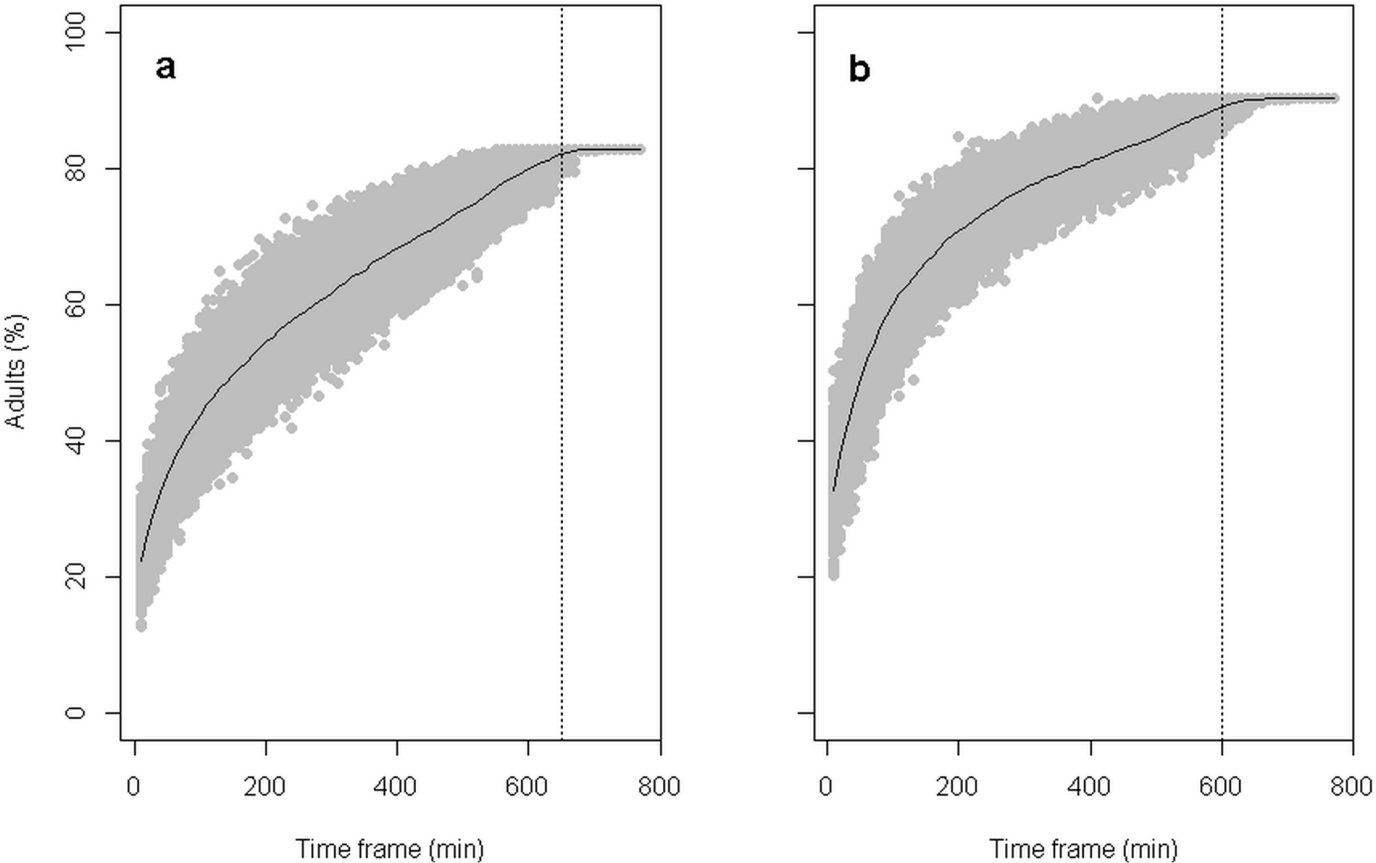
Percentage of the maximum number of adults observed in random time-frames of increased duration. Panels a and b show the mean percentages of the maximum number of adults observed in each warren in the three days of surveillance for all warrens and only for warrens with resident pups (active warrens), respectively. Vertical dotted lines indicate the shorter time frame when the percentage observed ranged within 0.05 of the asymptotic value.

The number of adults registered by minute was highly variable, with the maximum recorded only during short periods of time each day (Fig 3). Any sampling period much shorter than an entire day likely underestimates the number of adults associated with each warren. The shortest sampling period capable of registering 95% of the maximum number of adults from entire-day surveys was 650 minutes when considering all the warrens and 600 minutes when only warrens with pups were considered (Fig 4A and 4B). We did not find evidence of a diurnal trend in the visiting dynamics, because the fitted model did not show significant differences among the three periods of day tested (Table 2). The temporal variation in estimates of the number of adults associated with a warren caused by the estimation on different days was CV = 0.12, while the variation due to warren characteristics (random component) was CV = 0.45. Both variation components were increased when inactive warrens were considered for model fitting (temporal variation CV = 0.42; random component CV = 1.12).

**Table 2:** Diurnal trend in visiting dynamics.

	b_i_	S.E.	*t*_*16135*_	*P*
Morning (Intercept)	1.242	0.160	7.745	0.000
Noon	0.057	0.038	1.489	0.136
Afternoon	-0.004	0.052	-0.080	0.936

Estimated parameters for predictors of adult abundance comparing three times of day according to the fitted model. The informed estimated parameter (b_i_), standard error (S.E.), t statistic (*t*_*d*.*f*._) and associated probability (*P*) are given.

## Discussion

The two-step protocol was successfully applied to *D*. *patagonum* within the Península Valdés protected area, which was logistically suitable and allowed to warrens to be found and the associated adults to be counted, estimating detection probability. Both components of the two-step protocol revealed important sources of variation that could affect detectability. In this section, we discuss the results related to the different sources of variation and the alternative monitoring designs to cope with them.

Warren detectability was approximately 80%, with little variation, and the encounter rate was 0.14 km^-1^, much higher than the individual encounter rate of 0.05 km^-1^ (Section C in S1 File). These results confirm that it is more convenient to count warrens than individuals in the open field. The estimated detection probabilities showed that line transect sampling was effective for warren detection within sampled areas (searching plots), despite the fact that distance among consecutive transects (200 m) may seem too large in a shrubby landscape. In addition, given the little variation in detectability among landscape configurations, it could be possible to pool strata together, in which case the encounter rate could be high enough to apply distance sampling [4] to obtain the warren density and detection probability. Nevertheless, we acknowledge that our sample size (four sites) is small to make general statements and is conditioned to the species presence in the area. Thus, further investigation across the species range is needed to better understand the effect of landscape configurations over warren detectability and the limitations that the encounter rate could impose on sampling design and methodology.

False positives (misidentification and abandoned warrens) were much more important to accurate estimation than imperfect detection. Misidentification error could be reduced by more extensively training volunteer observers or by doing all searching with a smaller group of highly trained observers, which also would reduce the variation among observers. In both cases we still recommend re-checking (double sampling) warrens detected in a proportion of the searching areas to estimate misidentification error and include it in abundance estimations.

The largest source of error was abandoned warrens, which is functionally equivalent to the more common case of a suitable patch of habitat not being occupied by the target species. This source of variation could be included in the abundance estimation as the probability that a located warren has associated adults, randomly selecting warrens to observe with cameras and getting the proportion of active warren. However, the proportion of abandoned warrens is so high that counts in random sampled warrens would result in too many zero counts. This causes problems in abundance modelling, even assuming zero inflated distributions. The necessary effort to survey, select, and confirm that warrens are active is cost effective in terms of reducing the variability of counts, as discussed later.

Misidentification is a typical bias when the signs used are breeding or resting sites (e.g., mara warrens could be confused with peludo burrows or squirrel dreys with bird nests) and must be included in the protocol to avoid overestimations [16]. On the other hand, it is also important to not include abandoned sites and to understand the dynamic of their use [33]. Some rare species use the same breeding areas repeatedly and, therefore, are relatively easy to census; however, because some new nesting sites could be used each year, a sampling design should allow for its detection and census [33].

Our results show that surveillance cameras could replace an *in situ* observer to estimate the number of adults associated with a warren since we saw no differences in their detection capability. Similar results were observed in other species. For example, visual census and high definition video transects were compared for monitoring coral reef fish assemblages in marine ecosystems (e.g., [34]). Our results indicate that maras do not perceive the camera as a threat, and its presence would not affect the visiting behavior of adults to the warren. We saw two possible instances of reaction to the cameras (more distant individuals in warren 2 and more alert individuals in warren 12). These differences were not seen in the majority of warrens. In these two cases, the results may have been affected by other factors during observations, such as vehicular traffic in a road close to both warrens.

Other studies have also considered cameras a non-intrusive tool [19, 35, 36]. However, studies performing specific analyses to evaluate possible effects of the camera over individuals’ behavior are scarce. Although scarce, results seems to consistently show no negative effects, similar to our own findings; for example, beavers (*Castor canadensis*) did not show significant interactions with cameras and did not leave monitored warrens [35] and red foxes (*Vulpes vulpes*) showed a fast adaptation to the presence of cameras after initial negative effects in their behavior [37].

We found that samples shorter than a day (< 10 daylight hours) will underestimate the number of adults. This variation occurs because the maximum activity around warrens is short in duration, happens only a few times or just once during each considered day and is unpredictable because diurnal trends in visiting behavior are not evident. As a result, we do not recommend the use of movement- or time-triggered cameras for this species given that the probability of missing the maximum number of animals is high.

Repeated counts of the maximum number of adults registered simultaneously during a single-day observation period showed little variation, with a detection probability of approximately 90%. This modest amount of temporal variation, which would be associated with the continuous movement of adults around the monitored area, is part of the residual variation of the method. A similar result was reported in reef fish, given that the most relevant variation in fish counts was observed over a very short time period [13, 38]. This is clearly related to the high mobility of studied animals and with differences in the detection capacity of observers [38]. With the sampling design used here, it is not possible to differentiate short-term variation due to continuous movement from the methodological component of the variance related with the detection capacity of the camera.

Another source of temporal variation when counts are performed over temporary aggregations, such as around breeding sites, is related with the asynchrony of the individual breeding season. A single count on any day of the season underestimates the entire population, given that there is never a day when all individuals are present [39]. Although this source of variation was not assessed in our study, it has been studied in other cases, and models have been developed based on a few counts throughout the breeding period as well as independent evidence on the length of time individuals remain in an aggregation [39]. This information can be obtained from the scheme of the two-step protocol as proposed for repeating mara observations using cameras in the reproductive season. It can be possible to measure the permanence of couples using a warren by following the number of pups of different age classes [8] from a crèche born until they leave the warren (approximately six weeks old [14]).

The main variation in counts of adults was among warrens, once the temporal variation was reduced by taking observations over the entire day. The spatial variability was also mentioned as a key factor in birds counts that has to be included to correctly assess population trends [40]. This variation in the case of mara is not due to imperfect detection but to ecological processes. Previous studies have shown that the differences in the number of adults among warrens is related to the effects of environmental factors, such as vegetation and distance to anthropogenic structures, effects of socio-spatial interactions among warrens, the long term colonization dynamic, and site history in relation with sheepherding or hunting [8, 9, 15]. However, incorporating spatial variation using a stratified sampling design [33] is not yet possible because the importance and strength of those factors is not adequately quantified. Thus, if to estimate abundance it is necessary to take a sample of located warrens instead of performing counts in all of them, monitoring designs should include the largest number of warrens possible to improve the accuracy of the estimated mean number of adults that will be associated with all found warrens.

Temporal and among warren variation was increased when inactive warrens were included in estimations. Inactive warrens could be confused with active warrens due to the use of signs of mara presence (recently removed soil and fresh footprints or feces close by) that produce an incorrect assessment of warren status. Signs would be useful to recognize areas with maras [8], but not to identify reproductively active warrens, because many warrens are visited, but not all are used every year [14]. Some abandoned warrens can be discarded after several visits to search for new signs that should appear if it is active. If new signs appear, camera records are useful to confirm mara presence around the warren and also that they are breeding in it. It is important to confirm that pups are using the warren because only then will the adults consistently visit the warren. In addition, the occasionally sighted individual should not be included in estimations because they could be breeding in another warren, in which case they would be double counted, or they could be not breeding, and thus they would not be part of the target population.

The proposed method to estimate abundance is directed to the reproductively active portion of the population, which probably stays closer to the warren where they can be counted. Alternatively, abundance could be estimated without this bias using randomly arranged camera traps in the study area and modelling the process of contact between animals and the camera according to random encounter models [3]. However, given that the daily range of maras averages 1.7 km [9] and that estimated densities within our study area were lower than 1 km^-1^ [8], more than 1000 camera-days would be needed to obtain the minimum number of encounters required by the method [3]. In contrast, only 23 camera-days were needed to check and count breeding individuals within the study area.

Even though our two-step protocol resulted in estimates of detection and insight into potential pitfalls, it would not be wise to apply these estimates to other areas and times. The process of estimating detection probability and other source of variability could be incorporated in any monitoring protocol by double searching a proportion of the surveyed areas and double counting a proportion of the founded warrens [1]. Based on our results and following Pollock et al. [1] to calculate the allocation of sampling effort between collecting data on the count index and collecting the more detailed data to do the detectability estimation, we find that 22% of the surveyed areas should be double searched and 18% of warrens should be observed twice for the counting of adults. We believe that the application of this monitoring protocol over the mara range could provide reliable and systematic information about population abundance and spatial dynamics. Through the means of warren searches and surveillance with cameras within a given area, it is possible to obtain a minimum local population size and detect if there are new warrens, changes in the number of active warrens, or changes in the number of couples in each warren over a period of years. This information is critical for obtaining information about variation in the reproductively active population and long term trends in population abundance beyond this variation.

## Supporting Information

S1 Dataset Number of adults by minute recorded by the camera in each warren and sampling day. (XLS)

S2 Dataset Maximum number of adults recorded by the camera and the observer in each warren. (XLS)

S3 Dataset Number of alert adults, relaxed adults, adults distant from and close to the warren when cameras were present and when they were absent in each warren. (XLS)

S1 File Alternative methods and designs applied to mara within Península Valdés. Section A: Distance sampling. Section B: Random design to find signs of mara presence. Section C: Encounter rate of adult mara individuals in walking transects.
(DOC)
