# Supplementary material for: Sources of Variation in a Two-Step Monitoring Protocol for Species Clustered in Conspicuous Points: Dolichotis patagonum as a Case Study
Source: PLoS One. 2015 May 26;10(5):e0128133. doi: 10.1371/journal.pone.0128133 (PMC4444103; doi:10.1371/journal.pone.0128133)
Supplement: S1 File — Section A: Distance sampling. Section B: Random design to find signs of mara presence. Section C: Encounter rate of adult mara individuals in walking transects. (DOC) [file pone.0128133.s004.doc]

**Supporting Information S1**

Before the proposed two-step protocol was applied to the population of maras of Peninsula Valdés, other approaches including well established methods and random sampling designs were performed.

**Section A. Distance sampling**

During the abundance survey of guanaco (*Lama guanicoe*) performed in Península Valdés in November 2008, data on mara were collected as well. Line transect survey was conducted along 342 km of available dirt roads (Figure A). During that survey two observers standing in the back of a pick-up vehicle scanned the surroundings searching for animals. For every animal or group of animals encountered we stopped the vehicle and recorded group size. We also measured the distance and azimuth from the transect line to the location where the group was standing when detected, using a laser range finder, and registered the vehicle geographic location, using a global positioning system. Radial distances and azimuths recorded were used to estimate the perpendicular distance from the transect line to each group observed.

Results of the survey showed that only 22 individual or groups of mara were detected, giving an encounter rate of 0.07 km^-1^.

**Figure A**. Location of line transects for Distance samling (dark grey) and mara observations (black dots)

**Section B. Random design to found signs of mara presence.**

Random points were selected to place transects within Península Valdés using ArcView 3.2 (Figure B). Starting in these points we walked 2.5 kilometers searching for warrens and feces of mara. We found none warren after walking 22.5 km in nine random locations.

**Figure B**. Location of random transects within Península Valdés where sings of mara presence were searched.

**Section C. Encounter rate of adult individuals of mara.**

Adult mara sights were registered during the same line transects sampling performed to search for warrens described in Materials and Methods. Encounter rate (Table A) was calculated taking into account only records of trained observers to avoid false positive detections because of misidentification of european hare (*Lepus europaeus*) and mara. Given that each area was surveyed by a different group of observers, the distance over which encounter rate was calculated varies.

**Table A. Encounter rate of adult individuals of mara**.

| Survey area | A | B | C | D |
| --- | --- | --- | --- | --- |
| Number of adults registered | 1 | 5 | 2 | 1 |
| Distance walked by expert observers (km) | 40 | 50 | 30 | 50 |
| Encounter rate (km^-1^) | 0.025 | 0.1 | 0.067 | 0.02 |
